# *In Situ* Anabolic Activity of Periodontal Pathogens *Porphyromonas gingivalis* and *Filifactor alocis* in Chronic Periodontitis

**DOI:** 10.1038/srep33638

**Published:** 2016-09-19

**Authors:** Ralee Spooner, Kris M. Weigel, Peter L. Harrison, KyuLim Lee, Gerard A. Cangelosi, Özlem Yilmaz

**Affiliations:** 1 Divison of Periodontics, Department of Stomatology, Medical University of South Carolina, Charleston, SC 29425, USA; 2 Lieutenant, Dental Corps, Navy Professional Medicine Development Center, Bethesda, MD 20889, USA; 3 Department of Environmental and Occupational Health Sciences, University of Washington, Seattle, WA 98195, USA; 4 Department of Periodontology, University of Florida, Gainesville, FL 32610, USA; 5 Department of Oral Health Sciences, Medical University of South Carolina, Charleston, SC 29425, USA; 6 Department of Microbiology and Immunology, Medical University of South Carolina, Charleston, SC 29425, USA

## Abstract

*Porphyromonas gingivalis* and *Filifactor alocis* are fastidious anaerobic bacteria strongly associated with chronic forms of periodontitis. Our understanding of the growth activities of these microorganisms *in situ* is very limited. Previous studies have shown that copy numbers of ribosomal-RNA precursor (pre-rRNA) of specific pathogen species relative to genomic-DNA (gDNA) of the same species (P:G ratios) are greater in actively growing bacterial cells than in resting cells. The method, so-called steady-state pre-rRNA-analysis, represents a novel culture-independent approach to study bacteria. This study employed this technique to examine the *in situ* growth activities of oral bacteria in periodontitis before and after non-surgical periodontal therapy. Sub-gingival paper-point samples were taken at initial and re-evaluation appointments. Pre-rRNA and gDNA levels of *P. gingivalis* and *F. alocis* were quantified and compared using reverse-transcriptase qPCR. The results indicate significantly reduced growth activity of *P. gingivalis*, but not *F.* alocis, after therapy. The P:G ratios of *P. gingivalis* and *F. alocis* were compared and a low-strength, but statistically significant inter-species correlation was detected. Our study demonstrates that steady-state pre-rRNA-analysis can be a valuable culture-independent approach to studying opportunistic bacteria in periodontitis.

Periodontitis is a globally pervasive disease characterized by progressive destruction of tooth-supporting structures and affects millions of people^1^. Periodontitis is also associated with several chronic systemic diseases^2^. The composition of the dental biofilm is diverse and plays an important role in the development of periodontitis^3^,^4^. Organisms including *Porphyromonas gingivalis, Treponema denticola,* and *Tannerella forsythia* belong to the ‘red complex’ of the dental biofilm and have been established as contributors to periodontitis^5^,^6^,^7^,^8^. Disproportionate overgrowth of opportunistic bacterial pathogens, including the red complex organisms, couples with the host immune response to potentiate inflammation and destruction of the periodontium.

*P. gingivalis* is proposed to be a “keystone pathogen” due to the microorganism’s prominent role in modulating the dynamics of the host immune response and composition/structure of the dental biofilm in order to persist in oral tissues^9^,^10^,^11^,^12^,^13^,^14^. Another emerging opportunistic pathogen, *Filifactor* alocis, has lately become a subject of great interest due to its association with periodontitis^15^,^16^,^17^,^18^,^19^,^20^,^21^,^22^ and peri-implantitis^23^,^24^. Our current understanding of the interaction between *F. alocis* and other periodontal pathogens is limited, however recent evidence suggests the pathogenicity of *F. alocis* is likely potentiated by *P. gingivalis*^25^,^26^,^27^,^28^. Thus, investigating the role of *F. alocis* in periodontitis is warranted.

Culture-independent technologies, such as the Human Oral Microbiome Identification using Next Generation Sequencing (HOMINGS), have allowed us to better understand periodontitis by profiling the microbial composition of the dental biofilm with high specificity^29^,^30^. Limitations to our understanding persist however, despite the availability of increasingly sensitive technologies such as HOMINGS. Use of additional culture-independent as well as physiologically relevant molecular-based techniques could further characterize periodontitis and may help define the etiological roles played by certain bacterial species, such as *P. gingivalis* and *F. alocis*.

Ribosomal RNA precursor (pre-rRNA) represents a large percentage of total RNA within cells^31^. As cell growth diminishes, pre-rRNA synthesis halts while maturation of the precursor molecules continues, resulting in substantial depletion of the pre-rRNA pool^32^,^33^. The pre-rRNA pool rapidly recovers upon nutritional stimulation^34^,^35^,^36^. Because pre-rRNA sequences are species-specific, it is possible to detect and quantify pre-rRNAs of specific bacterial species by applying reverse transcriptase quantitative PCR (RT-qPCR) to complex clinical and natural samples. Ratiometric pre-rRNA analysis, also termed molecular viability testing (MVT), has been shown to efficiently assess the viability of microbial pathogens in environmental and patient samples through detection of the synthesis of pre-rRNA upon brief nutritional stimulation^34^,^35^,^37^. MVT has also demonstrated enhanced sensitivity to the presence of viable microorganisms compared to DNA-targeted qPCR^36^.

Steady-state pre-rRNA analysis, a separate but related culture-independent method, assesses the growth activity of bacteria in samples without nutritional stimulation. Studies have shown that pre-rRNA copy number correlates with growth activity of bacterial cells (actively dividing cells have a high pre-rRNA copy number due to ongoing rRNA synthesis)^32^,^33^,^38^. Therefore, measurement of species-specific pre-rRNA normalized to genomic DNA (gDNA) of the same species (P:G ratio) can be used to assess ongoing proliferation of the targeted species in environmental or clinical samples. Due to the successful application of molecular methods similar to steady-state pre-rRNA analysis in other settings, we believe that this approach could be a valuable for understanding the growth activity/behavior of bacterial pathogens in periodontitis.

In this study we applied steady-state pre-rRNA analysis as a novel culture-independent approach to assess bacterial growth activity in periodontitis. We quantified pre-rRNA and gDNA of *P. gingivalis* and *F. alocis* from clinical chronic periodontitis samples before and after non-surgical periodontal therapy using RT-qPCR. P:G ratios were then generated to compare growth activity of *P. gingivalis* and *F. alocis* at baseline and after therapy. Clinical parameters including probing pocket depth (PPD), bleeding on probing (BOP), and presence of supra-gingival plaque were recorded before and after treatment to test for associations with growth activity as reflected in P:G ratio. Finally, the P:G ratios of *P. gingivalis* and *F. alocis* were compared to determine if any inter-species relationships could be found.

## Results

### Clinical findings after non-surgical periodontal treatment

Fifteen patients were enrolled in this study. Clinical diagnosis of periodontitis was completed using the guidelines of the American Academy of Periodontology^39^. Based on PPD and clinical attachment level (CAL), the periodontal pockets with the deepest PPD were selected as sample collection sites. A total of 45 sites were assessed for growth activity of *P. gingivalis* and *F. alocis* at initial and re-evaluation appointments using steady-state pre-rRNA analysis.

All sample sites had PPD ≥ 4 mm prior to treatment and 24 sample sites had PPD ≥ 4 mm upon re-evaluation. The PPDs were significantly reduced (one-tailed paired *t*-test, p = 2.5 × 10^−13^) from 5.82 ± 1.17 mm to 4.00 ± 1.45 mm after treatment. Mean improvement of CAL was 3.09 ± 2.65 mm after treatment (one-tailed paired *t-*test, p = 3.8 × 10^−8^), Table 1. Thirty-nine sample sites displayed BOP at baseline and 18 sample sites showed BOP at re-evaluation. The reduction in BOP after treatment was significant (McNemar’s test, p = 6.3 × 10^−5^). Forty sample sites had supra-gingival plaque at initial evaluation and 18 sample sites had supra-gingival plaque at re-evaluation. The reduction in plaque detected was significant (McNemar’s test, p = 4.5 × 10^−6^).

### Measurement of actively growing bacteria by steady-state pre-rRNA analysis

Because pre-rRNA and gDNA are not measured with equal efficiency (RT-qPCR is generally less efficient than qPCR), this value does not indicate that resting bacterial cells have on average, fewer than 0.5 or 0.1 pre-rRNA molecules per genome. The actual number of pre-rRNA molecules in resting cells is not known. However, by applying the same measurements at initial sample collection and re-evaluation appointments, it was possible to generate comparative reliable results.

**Figure 1:**
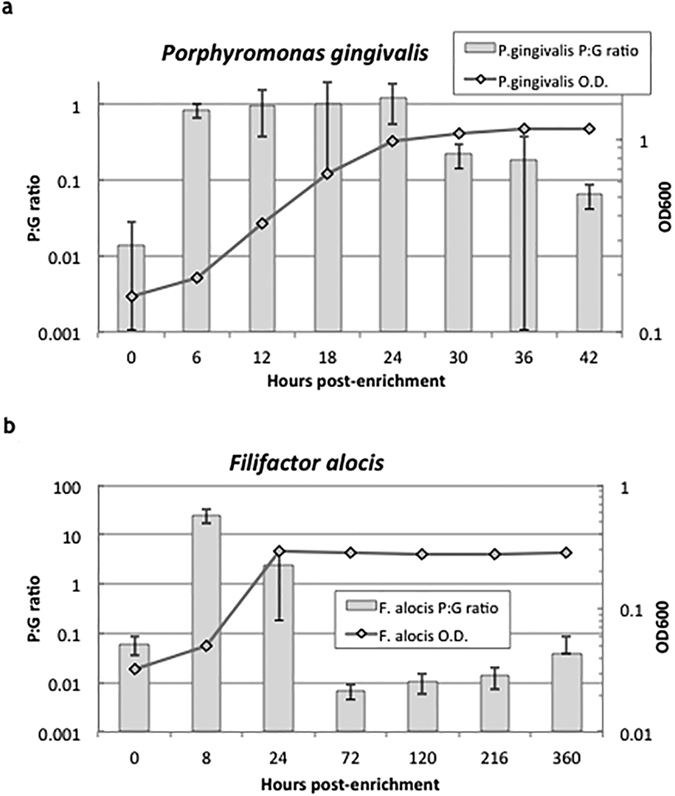
Growth curves of *P. gingivalis* and *F. alocis* used to generate pre-rRNA:genomic-DNA (P:G) ratios. (**a**) Growth curve of *P. gingivalis* over 42 h time course. At inoculation and 6 h intervals, samples of the culture were taken and the P:G ratios were measured using RT-qPCR. The P:G threshold value of 0.5 was determined by identifying a point in the stationary phase that correlates with the highest P:G ratio. Thus, the threshold P:G ratio represents a value above which the bacteria can be conservatively viewed as actively dividing and metabolically active. (**b**) Growth curve of *F. alocis* over 360 h time course. P:G ratios were measured at time points and the threshold value of 0.1 was selected following the same strategies applied to *P. gingivalis.*

Threshold values delineating growing vs. non-growing cell populations were derived from laboratory-based growth curve experiments, which identified the maximum P:G ratios that were seen in all samples of non-growing cultures (Fig. 1). Thresholds selected for this study were 0.5 for *P. gingivalis* and 0.1 for *F. alocis*. These values corresponded to the highest P:G ratios observed during the log phase of the bacterial growth curves from our nutrient-depletion experiment (see Methods). Use of alternative threshold values did not significantly alter results and interpretations.

### Association between clinical parameters and actively growing *P. gingivalis* and *F. alocis*

The relationships between actively growing bacteria, as determined by P:G ratio, and clinical observations were determined using logistic regression. Actively growing *P. gingivalis* was positively correlated with PPD ≥ 4 mm before (p = 0.04) and after (p = 0.009) treatment. A significantly positive correlation (p = 0.0001) between actively growing *F. alocis* and PPD ≥ 4 mm was found only in sample sites after treatment.

Actively growing *F. alocis* was found to be positively correlated with BOP in sample sites at initial collection (p = 0.005), but not at re-evaluation. No significant associations between actively growing *P. gingivalis* and BOP or plaque score were found in our analysis. Positive associations between actively growing *F. alocis* and presence of plaque were found before (p = 0.003) and after treatment (p = 0.03).

### Prevalence of actively growing *P. gingivali*s and *F. alocis* after non-surgical periodontal therapy

**Figure 2:**
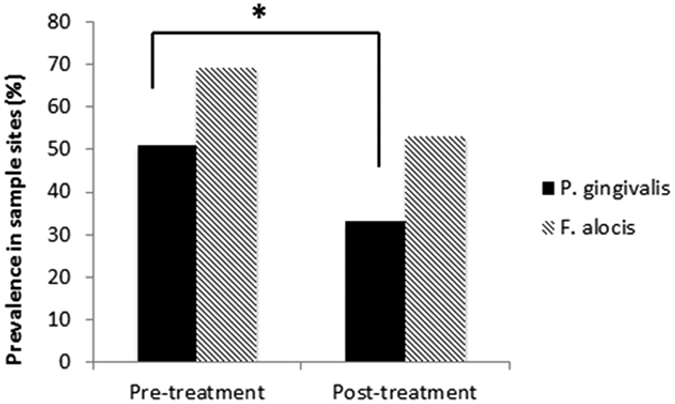
Prevalence of actively growing periodontal pathogens in periodontitis sample sites. Percent of disease sample sites with actively growing *P. gingivalis* and *F. alocis* at initial and non-surgical periodontal therapy re-evaluation appointments (Logistic regression, *denotes statistical significance, p < 0.05).

The number of sample sites with actively growing *P. gingivalis* and *F. alocis* was quantified at initial collection appointments and at re-evaluation (Fig. 2). At initial collection, 51% and 69% of sample sites had actively growing *P. gingivalis* or *F. alocis*, respectively (Fig. 2). After treatment, 33% and 53% of sample sites had actively growing *P. gingivalis* or *F. alocis*, respectively. The reduction in prevalence of *P. gingivalis* (Logistic regression, 95% CI: 0.2535, 0.7633) was statistically significant. The prevalence of actively growing *F. alocis* was also reduced, but the reduction was not significant (Logistic regression, 95% CI: 0.4750, 0.9315). Thirteen and twenty four sample sites with actively growing *P. gingivalis* or *F. alocis,* respectively, were observed at both initial collection and re-evaluation appointments.

### Changes in bacterial load determined via genomic DNA quantification

**Figure 3:**
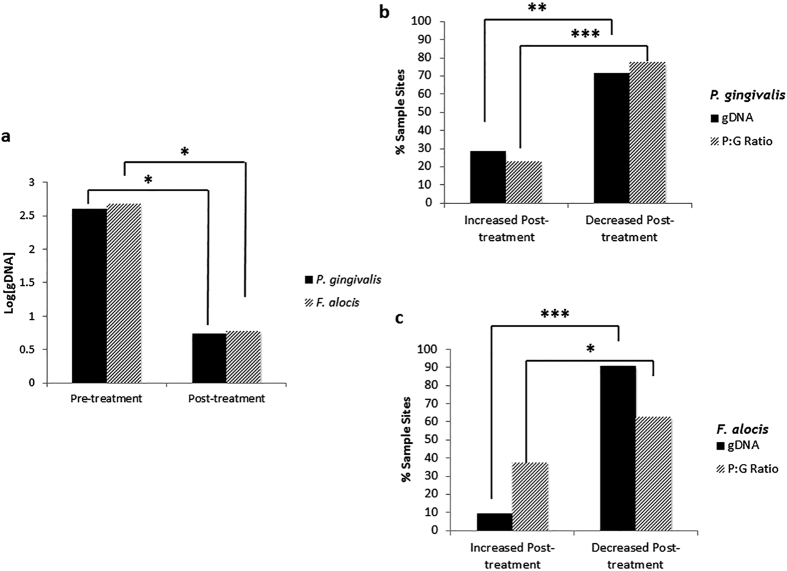
Changes in bacterial load and activity level of *P. gingivalis* and *F. alocis.* (**a**) Levels of gDNA for *P. gingivalis* and *F. alocis* at initial and non-surgical periodontal therapy re-evaluation appointments. [gDNA] was measured in μg and was log-transformed for easier interpretation of data. (Student’s *t-*test, *denotes statistical significance at p < 0.001). (**b**) Percent of total sites in which *P. gingivalis* [gDNA] or P:G ratio increased or decreased after treatment. (Two-sample *Z*-test for proportions, *denotes statistical significance at p < 0.05; **denotes statistical significance at p < 0.01, ***denotes statistical significance at p < 0.0001) (**c**) Percent of total sites in which *F. alocis* [gDNA] or P:G ratio increased or decreased after treatment. (Two-sample *Z*-test for proportions, *denotes statistical significance at p < 0.05; **denotes statistical significance at p < 0.001).

The bacterial load of *P. gingivalis* and *F. alocis* was indirectly determined via species-specific gDNA quantification before and after treatment (e.g. changes in [gDNA]) (Fig. 3). *P. gingivalis* and *F. alocis* load decreased significantly (Student’s *t*-test, p = 2.1 × 10^−5^ and p = 1.1 × 10^−6^, respectively) after treatment (Fig. 3a). We also evaluated the proportion of sites exhibiting growth activity and bacterial load changes for *P. gingivalis* (Fig. 3b) and *F. alocis* (Fig. 3c). There was a significantly higher proportion of sites in which the [gDNA] decreased for *P. gingivalis* (Two-sample Z-test, *Z* = −3.14, p = 0.002) and *F. alocis* (Two-sample *Z*-test, *Z* = −4.52, p = 6.3 × 10^−6^). Similarly, there was a significantly higher proportion of sites in which the P:G ratio decreased for *P. gingivalis* (Two-sample Z-test, *Z* = −6.48, p < 0.0001) and *F. alocis* (Two-sample *Z*-test, *Z* = −2, p < 0.05).

### Correlating growth activity between *P. gingivalis* and *F. alocis*

**Figure 4:**
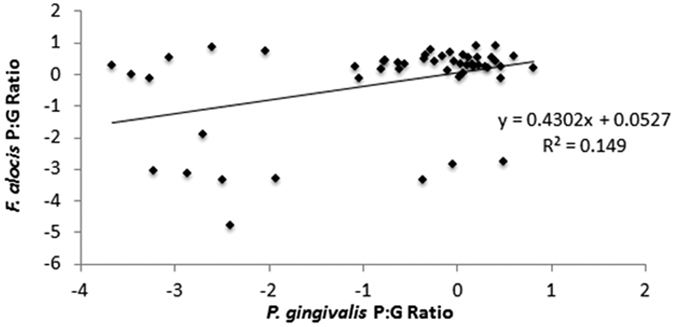
Correlation of growth activity of *P. gingivalis* and *F. alocis.* Correlation of *P. gingivalis* and *F. alocis* P:G ratios using linear regression (combined initial and non-surgical periodontal therapy re-evaluation appointments). Trendline equation: log(*F. alocis* P:G ratio) = 0.4302 * log(*P. gingivalis* P:G ratio) + 0.0527. R^2^ value: 0.14902. Outliers (greater than 3 standard deviations from the mean) and sample sites in which P:G ratios of only one target organism was generated were removed from analysis. Number of samples (sites with P:G ratios for both microorganisms) included in analysis = 51.

The growth activities of *P. gingivalis* and *F. alocis* relative to one another were assessed. Sample sites in which the P:G ratio of only one target organism was detected and outlier P:G ratios (greater than three standard deviations from the mean) were removed from this analysis (samples included in analysis *n* = 51). P:G ratios were log-transformed and compared using linear regression (Fig. 4). A small strength, but statistically significant correlation was observed (Pearson’s correlation coefficient r = 0.39, p = 0.005).

## Discussion

This study investigated the use of steady-state pre-rRNA analysis to assess the growth activity of the periodontal pathogens *P. gingivalis* and *F. alocis* in patients with chronic periodontitis. Our results suggest that this novel culture-independent technique could be useful for directly understanding bacterial growth activities. This is the first study to evaluate the *in situ* growth activity of periodontal pathogens in patients with periodontitis through culture-independent techniques.

Non-surgical periodontal therapy provided to the study population resulted in improvement in clinical parameters associated with periodontitis and lowered the prevalence of actively growing *P. gingivalis*. Previous studies have shown a decrease in the relative abundance^8^,^20^,^40^ and number of viable cells^41^ of *P. gingivalis* following non-surgical periodontal therapy, thus our findings are consistent with existing literature.

Interestingly, we did not observe a significant reduction of actively growing *F. alocis* following non-surgical periodontal therapy. *F. alocis* has been previously implicated in cases of refractory periodontitis^18^ and non-surgical periodontal therapy without the use of antibiotics has shown limited effectiveness on reducing *F. alocis*^42^. Additionally, evidence suggests that the use of antibiotics may not have as great an effect on response to non-surgical periodontal therapy compared to the composition of the oral microbiota prior to treatment^43^. More studies are needed to determine the effectiveness of antibiotics on actively growing *F. alocis in situ,* and steady-state pre-rRNA analysis could be useful in such studies.

We compared the total load using [gDNA] of *P. gingivalis* and *F. alocis* at initial and re-evaluation appointments. Our results show a significant decrease in the abundance of these organisms. More comprehensive techniques, such as the Human Oral Microbiome Identification Microarray (the pre-cursor to HOMINGS), have also supported this result^8^,^20^. In order to assess the comparability of changes in bacterial growth activity and load, the increase/decrease in P:G ratios versus the [gDNA] was recorded. The two methods of measurement offered comparable results, however steady-state pre-rRNA analysis could provide a more detailed picture of bacterial growth *in situ* rather than relying solely on detecting nucleic acid.

One aim of this study was to identify any relationship between the growth activities of *P. gingivalis* and *F. alocis* before and after non-surgical periodontal therapy. The dental biofilm is a highly complex, multi-level ecosystem in which constituent bacterial species compete for nutrients and potentiate disease^4^. The specific interaction of *P. gingivalis* and *F. alocis* is a newly studied phenomenon and the growth of one relative to another may be a key indicator of conditions within the dental biofilm^28^. In this study a statistically significant, albeit weak, positive association was found between the P:G ratios of *P. gingivalis* and *F. alocis*. To date, only *in vitro* models have been used to investigate the potential interaction of *P. gingivalis* and *F. alocis*^25^,^26^,^27^. Thus, our study may offer a glimpse into a physiological and environmental relationship between these two opportunistic pathogens.

The relative amount of oral bacteria, including *P. gingivalis* and *F. alocis*, and their presence in patients with varying degrees of health and disease has become an active area of research over the past few years^30^,^44^,^45^. Our investigation originally aimed to identify healthy matched sample sites, however we faced challenges in identifying sample sites with no clinical signs of inflammation in this study population. Furthermore, the present study did not account for patient smoking status. In any future applications of steady-state pre-rRNA analysis in periodontitis research, it could be beneficial to include this demographic, as it is clinically and ecologically relevant^46^. We also included patients with varying degrees of chronic periodontitis and it may be interesting for further studies to select a more homogeneous patient population.

In conclusion, we applied steady-state pre-rRNA analysis as a culture-independent technique to assess growth activity of *P. gingivalis* and *F. alocis* in patients with chronic periodontitis. Our results demonstrate the potential value of this novel tool in the study of periodontitis. In future studies our approach can be expanded to larger patient populations using additional treatment modalities to further validate these new findings. Furthermore, it would be gainful to include other oral bacteria in future studies, with the aim to further characterize the dynamic nature of the dental biofilm in health and disease.

## Materials and Methods

### Study population

The clinical assessment and sample collection components of this longitudinal non-randomized cross-disciplinary study was performed on patients in the teaching clinics of the University of Florida College of Dentistry (UFCD) in Gainesville, Florida under the approved guidance of the Health Science Center Institutional Review Board (IRB, human subjects assurance number FWA 00005790) with all applicable federal regulations governing the protection of human subjects. Written informed consent was obtained from all subjects. The UFCD and the University of Washington IRBs also approved all experimental protocols. Molecular microbiological analyses were completed at the UFCD and University of Washington, in Seattle, Washington. Partially or fully dentate patients with at least twenty teeth were recruited. The patient age range for enrollment spanned the ages of 21 and 60 years, and the patient median age was 48 ± 11.5. Exclusion criteria for this study, in accordance with criteria outlined by the American Academy of Periodontology (AAP)^39^, included diagnosis with any systemic disease that could influence the progression and/or clinical characteristics of periodontal disease. Patients taking medications, which may affect the oral presentation of the periodontium or cause immune-modulating effects, were also excluded. These included antibiotics within three months prior to study enrollment, corticosteroids, chemotherapeutic agents, and bisphosphonates. Smoking status was not collected from patients. No exclusions were made on the basis of gender or race. However, our study population included 8 females and 7 males, for a total of 15 study participants, thus an approximately equal representation of the sexes was enrolled.

### Clinical examination and sample site selection

Full periodontal charting of the patients’ dentition was recorded, including probing pocket depth (PPD, measurement from gingival margin to depth of pocket), clinical attachment level (CAL, measurement from cement-enamel junction to depth of pocket), plaque (presence or absence), and bleeding on probing (BOP, presence or absence). Measurements were preformed using UNC-12 periodontal probes (Hu-Friedy, Chicago, IL, USA). Diagnosis of the patients’ periodontal clinical presentation was made in accordance with criteria outlined by the AAP^39^. Ten study participants were diagnosed with generalized severe chronic periodontitis and one diagnosis each of generalized moderate or generalized slight chronic periodontitis was made. One diagnosis each of localized slight, localized moderate, and localized severe chronic periodontitis were also made. Two anterior, one pre-molar, and 11 molars (including one third molar) were selected as sample sites at initial evaluation appointments. The criteria used to select these sample sites included periodontal pockets with the deepest clinical PPD and worst CAL. Presence of BOP and plaque was not a requirement for sample site selection.

### Periodontal therapy

Non-surgical periodontal therapy was completed after initial sample collection in the UFCD periodontology clinics. This therapy included supra- and sub-gingival debridement via scaling, root planing, and oral hygiene instructions. Re-evaluation was completed at least 6 weeks post-treatment by the same provider/trained periodontist. The study patients received supra- and sub-gingival scaling and oral hygiene instructions and were recommended to return for 3-month supportive periodontal maintenance.

### Bacterial growth curve generation using pre-rRNA upshift protocol

Ratios of pre-rRNA to gDNA (P:G ratios) were measured for both species to assess their respective growth activities. Threshold P:G ratio values to discriminate growing from non-growing populations were identified based on growth curves created for *P. gingivalis* and *F. alocis* (Fig. 1). *P. gingivalis* ATCC 33277 was cultured anaerobically for 36 h at 37 °C in trypticase soy broth (TSB) (Fisher Scientific, Waltham, MA, USA) supplemented with yeast extract (1 mg/mL) (Fisher Scientific), haemin (5 μg/mL) (Sigma-Aldrich, St. Louis, MO, USA) and menadione (1 μg/mL) (MP BIomedicals, Santa Ana, CA, USA). At 36 h, the culture of *P. gingivalis* was re-inoculated at 1:10 dilution into fresh supplemented TSB and grown at 37 °C for one week to reach late stationary phase and allow for drainage of the pre-rRNA pool. 100 μL of the carbon-depleted culture was harvested in triplicate and centrifuged at 6000 g and 4 °C for 10 min. The pellets were immediately placed onto ice and transferred to −80 °C for storage. The carbon-depleted culture was then re-inoculated at 1:10 dilution into pre-warmed fresh supplemented TSB and grown at 37 °C. At 6, 12, 18, 24, 30, 36, and 42 h post-inoculation 1 mL samples were harvested according to the methods described above.

The growth curve used to generate the threshold P:G ratio for *F. alocis* was made via the same approach to that of *P. gingivalis. F. alocis* ATCC 35896 was cultured anaerobically for 36 h at 37 °C in brain heart infusion (BHI) (Fisher Scientific) supplemented with yeast extract (0.5 mg/mL) (Fisher Scientific), L-cysteine (50 μg/mL) (Sigma-Aldrich), and 20% arginine (Fisher Scientific). At 36 h, the culture of *F. alocis* was re-inoculated at 1:10 dilution into fresh supplemented BHI and grown at 37 °C for one week to reach late stationary phase and allow for drainage of the pre-rRNA pool. Pelleted samples were collected according to the methods described above at 0, 8, 24, 72, 120, 216, and 360 post-carbon depletion.

### Bacterial sampling protocol

Teeth with periodontal pockets selected for sampling were isolated with cotton rolls and air dried to prevent saliva contamination. Sub-gingival plaque collection was completed using the paper point collection technique, which has been found to be similar to curette sampling in its ability to detect specific bacteria via qPCR^43^. Paper points (Henry-Schein, Melville, NY, USA) were inserted into the periodontal pockets and held *in situ* for at least 20 seconds. The paper points were then placed in sterile Eppendorf tubes which were directly put in cooler boxes previously frozen at −80 °C (Nalgene, Fisher Scientific). Upon collection, frozen boxes with samples were immediately stored and kept at −80 °C for future processing.

### Nucleic acid extraction and RT-qPCR

DNA and RNA of each individual sample were simultaneously extracted using the MasterPure Complete DNA and RNA Purification Kit (Epicentre, Madison, WI, USA). Paper points were suspended in the provided lysis buffer (300 μL and proteinase k solution (1 μL) prior to following the furnished protocols. The suspension was vortexed for 1 minute and heated at 65 °C for 15 minutes to lyse the cells. Total nucleic acid (TNA) was eluted in 30 μL TE, from which 10 μL was removed for DNA measurement. From the remaining 20 μL, RNA was purified by DNase I treatment and re-precipitated as directed by the included protocol, and resuspended in the same volume of RNAse-free water.

Pre-rRNA and purified DNA was directly measured by qPCR as follows. All primers were designed using Primer3 software. qPCR primers for *P. gingivalis* were F- CGAGGTGTACTACCTGATAAATCG and R- CCCTCGACTTGCATGTGTTA and the RT primer was GTTTCAACGGCAGGCTGA. qPCR primers for *F. alocis* were F- AACCGGAGCAAAACTGAGAA and R- CCGTCCGCCACTAACTTCTA and the RT primer was TACTGATCGTTGCCTTGGTG. As in previous studies^35^,^36^,^37^, RT-qPCR primers for amplifying pre-rRNA straddled the junction between the 5′ terminus of the mature rRNA (16S) and the pre-rRNA leader region (ETS1), such that intact pre-rRNA molecules were required as templates. An *in silico* analysis of the primer sequences using NCBI’s BLAST tool suggested that all four 16S paralogs would be detected for every published complete genome strain of *P. gingivalis* (n = 7) and *F. alocis* (n = 1). A similar analysis using NCBI’s BLAST tool against the non-redundant database predicted no non-target amplification.

RNA was converted to cDNA by reverse transcription (RT) with the ImProm II system (Promega, Fitchburg, WI, USA), using 4 μL template and 3 μM 16S-specific primers. Genomic DNA and cDNA were measured by qPCR (using the same primer/probe set) utilizing Power SYBR Green (Life Technologies, Grand Island, New York, USA). Each 20 μL reaction contained 2 μL template and 375 nM each of forward and reverse primers. Reactions (including 7-log standard curves) were run on an Applied Biosystems StepOnePlus under the following conditions: 95 °C for 10 minutes followed by 40 cycles of 95 °C for 15 seconds, 57 °C for 30 seconds, and 72 °C for 30 seconds. Thresholds were automatically set by the StepOnePlus software (Life Technologies, Grand Island, New York, USA) or manually adjusted in the exponential range when necessary.

### Statistical analyses

Differences in microbiological and clinical parameters were assessed using Logistic regression, Linear regression, Student’s *t*-test, and McNemar’s test. Logistic regression was completed with Statistical Analysis System GENMOD procedure using generalized estimating equation models for both binary and continuous outcomes. 95% confidence intervals were generated based on frequency tables created to compare prevalence of actively growing *P. gingivalis* and *F. alocis* before and after treatment. Log-transformation of data was completed when necessary to allow for interpretation of data. Linear regressions were performed using Excel 2010.

## Additional Information

**How to cite this article**: Spooner, R. *et al.* *In Situ* Anabolic Activity of Periodontal Pathogens *Porphyromonas gingivalis* and *Filifactor alocis* in Chronic Periodontitis. *Sci. Rep.* **6**, 33638; doi: 10.1038/srep33638 (2016).

## Figures and Tables

**Table 1:** Summary of clinical findings.

Clinical parameter	Pre-treatment	Post-treatment
PPD ≥ 4 mm	45 (100)	24 (53)
Average PPD	5.82 ± 1.17 mm	4.00 ± 1.46 mm*
BOP	39 (87)	18 (40)^§^
Plaque	40 (89)	17 (38)^§^
Average CAL	7.27 ± 2.16 mm	4.18 ± 1.87 mm*

Clinical parameters including the gender, number of pockets with PPD ≥ 4 mm, the average PPD, bleeding index (present/absent), and plaque index (present/absent) were assessed for all sites pre- and post-treatment. Values given are shown as number (percent of total sites). The significance in reduction of clinical signs of inflammation after treatment was assessed (*Paired *t*-test, p < 0.0001. ^§^McNemar’s test, p < 0.0001).
